# Optimization of UWB Base Station Deployment for Formwork Scaffolds in Underground Construction with Sub-Meter Positioning Accuracy by Semi-Controlled Field Experiments

**DOI:** 10.3390/s26041340

**Published:** 2026-02-19

**Authors:** Gang Yao, Lang Liu, Yang Yang, Xiaodong Cai, Xin Yang, Huiwen Hou, Mingpu Wang, Pengcheng Li

**Affiliations:** 1 School of Civil Engineering, Chongqing University, Chongqing 400045, China; yaogang@cqu.edu.cn (G.Y.); 202416131378@stu.cqu.edu.cn (L.L.); 202316131156t@stu.cqu.edu.cn (X.C.); 202516131193t@stu.cqu.edu.cn (X.Y.); 202316131167t@stu.cqu.edu.cn (H.H.); lipengcheng@cqu.edu.cn (P.L.); 2 State Key Laboratory of Safety and Resilience of Civil Engineering in Mountain Area, Chongqing 400045, China; 3 Department of Civil Engineering, Tsinghua University, Beijing 100084, China; wangmingpu@mail.tsinghua.edu.cn

**Keywords:** UWB positioning, construction safety monitoring, base station deployment, formwork scaffold, experimental optimization, 3D positioning

## Abstract

Fall-from-height fatalities in underground construction are closely associated with formwork scaffold operations, where dense steel members cause severe non-line-of-sight (NLOS) and multipath effects that degrade positioning performance. Although ultra-wideband (UWB) technology offers high theoretical ranging accuracy, its deployment-dependent performance in metal-rich scaffold environments remains insufficiently quantified. This study focuses on physical deployment optimization rather than algorithmic compensation. A full-scale formwork scaffold was constructed, and a stepwise one-factor controlled experimental design was employed to quantify the effects of anchor height (*H*) and horizontal spacing (*S*) on 3D positioning accuracy. The results show that sub-meter accuracy can be achieved through appropriate deployment, with a minimum 3D RMSE of 0.317 m and over 80% of single-axis errors confined within a 0.2 m engineering-valid region. For this specific setup, the optimal *S* = 1.5 m correlates with the scaffold grid size (approximately 0.8 times the 1.8 m bay width). While we hypothesize this ratio dependency applies to other geometries, this remains a site-specific observation requiring future cross-validation. Further analysis indicates that this deployment balances vertical signal visibility and multipath suppression. In addition, while the Position Dilution of Precision (PDOP) metric reflects geometric sensitivity, it does not linearly correlate with actual positioning errors under coplanar UWB deployments. These findings provide a rigorous static error model, serving as a critical prerequisite for developing robust real-time safety monitoring systems in scaffold-intensive construction environments.

## 1. Introduction

Fall-from-height fatalities represent a critical safety concern in the construction industry. A significant percentage of fatal work-related injuries stem from falls from height. For example, in the U.S. construction industry, 18% of fatal accidents were associated with such falls, while in non-fatal accidents within the Portuguese construction industry, falls from height accounted for 20%. These accidents tend to concentrate in medium and low-height work scenarios (below 9 m), with new commercial projects and low-cost residential projects being typical high-risk areas. Small-scale construction sites, due to budget constraints and inadequate safety measures, bear nearly 50% of fall accidents. These sites often feature weak safety management, underscoring the urgent need for real-time safety monitoring of workers performing high-altitude operations. Accurate positioning of workers and timely warnings can effectively reduce the occurrence of such accidents [1].

Positioning technology is critical for personnel safety monitoring within complex engineering and construction sites. However, technologies such as the global positioning system (GPS) experience significant accuracy declines in indoor environments due to wall obstructions, multipath effects, and interference, failing to meet real-time precision requirements [2]. In tests at high-risk locations such as power substations, researchers found that GPS is nearly ineffective in indoor, equipment-dense areas. By contrast, the UWB positioning system significantly enhances reliability because of its strong resistance to multipath interference [3]. In high-risk scenarios like bridge construction, UWB positioning technology enables real-time acquisition of 3D coordinates of personnel and vehicles and, when combined with dynamic building information modeling (BIM) visualization and hazard warnings, has been shown at underground garage construction sites to significantly improve the safety of high-altitude work [4].

UWB technology offers high temporal resolution, strong resistance to multipath interference, and the potential for reliable engineering accuracy. Collectively, these advantages make it a key approach for positioning personnel in complex construction environments [5]. Furthermore, characterized by low cost and minimal power consumption, UWB systems stand out in practical applications. Their ultra-wideband signals also have strong anti-interference capabilities and high spatial resolution, which ensure reliable communication and positioning in complex environments. These advantages position UWB as a crucial technical direction for addressing the challenge of real-time positioning for construction workers in complex settings such as underground spaces, indoor environments, and reinforced concrete structures. For instance, researchers demonstrated that a UWB system could achieve a 3D positioning error of less than 35 cm in 25–50 m^2^ indoor areas [6]. They proposed a low-cost scheme using commercial smartphones and UWB, providing a practical solution for safety management in resource-constrained scenarios.

In formwork scaffolding construction scenarios, UWB positioning faces significant additional challenges. The dense steel tubing and complex spatial structures of formwork scaffolds create unique obstruction patterns, which cause severe NLOS propagation and multipath interference. These effects disrupt UWB signal propagation and degrade positioning accuracy. Although filtering algorithms can mitigate errors from multipath effects, a more fundamental challenge is the timing disruption caused by NLOS propagation itself, which leads to a sharp decline in time-of-arrival (TOA)/time-difference-of-arrival (TDOA) positioning accuracy. Specifically, in NLOS conditions, UWB signals may either penetrate obstacles and incur additional delays or be reflected along longer paths. Both scenarios cause an overestimation of distances and ultimately result in significant positioning errors [7].

Existing review studies have systematically organized the association between real-time locating systems (RTLS) and personnel safety management in construction scenarios. These studies clearly indicate that UWB technology, due to its characteristics of strong resistance to multipath interference and high positioning accuracy, has become a key technical direction for addressing the challenge of personnel positioning in complex construction environments. Conventional filtering algorithms, such as the standard Kalman filter (KF), often exhibit significant performance degradation in metal-rich environments due to their inherent sensitivity to non-Gaussian noise and outliers induced by severe multipath and NLOS conditions [8]. Regarding these issues, a large body of existing research has proposed algorithm optimization strategies. NLOS errors are mitigated using methods such as joint filtering, multipath identification, or deep learning. Among these, the cascaded KF method proposed by researchers significantly enhances positioning accuracy even under long-term NLOS conditions [7]. Additionally, numerous studies have integrated data from inertial measurement units (IMU) and visual sensors into UWB positioning systems to improve robustness and expand positioning functions. In safety monitoring scenarios, these algorithm optimizations provide a more reliable performance foundation for UWB systems. Nevertheless, the literature also emphasizes that while most studies focus on algorithm optimization in general scenarios, there remains a significant lack of systematic exploration regarding the independent and interactive effects of base station deployment parameters (e.g., height, spacing) on positioning accuracy in strong NLOS scenarios with dense metal components, such as formwork scaffold environments [9].

In practical industrial and construction environments, obstacles such as walls, equipment, and personnel, along with complex spatial configurations, cause NLOS propagation and multipath effects that severely degrade UWB positioning performance. Research on positioning in small-scale scenarios shows that the impact of the spatial geometric layout between base stations and mobile terminals (MT) on 3D positioning accuracy is significantly greater than the difference between different least squares algorithms. Through experiments, the study further found that increasing the height difference between base stations and mobile terminals can effectively reduce the dilution of precision (DOP) value, thereby significantly minimizing positioning errors [6]. High-precision positioning within 0.5 m can be achieved in complex construction scenarios with handover issues by appropriately reducing the number of positioning base stations to gain a higher observation frequency [10]. Additionally, researchers conducted experimental studies on multiple deployment schemes and found that the accuracy of UWB systems is extremely sensitive to deployment schemes, with positioning accuracy fluctuating significantly as deployment methods change [11]. Therefore, in complex NLOS environments, while the design of positioning algorithms is certainly important, the physical optimization of base station deployment parameters is equally critical.

This study aims to investigate the impact of physical deployment geometry on UWB positioning accuracy in metal-rich formwork scaffolds, and to explore the optimal base station deployment parameters to establish a static accuracy baseline. This baseline is intended to isolate structural multipath effects from dynamic noise, laying a theoretical foundation for future dynamic trajectory tracking. Section 2 elaborates on the theoretical foundations and error mechanisms of UWB 3D positioning, analyzes the limitations of the traditional PDOP evaluation system in coplanar base station layouts, and provides a theoretical basis for subsequent deployment optimization. Section 3 details the design of the experimental scheme, including the construction of the simulated formwork scaffold structure and the multimodal data collection methods. Section 4, based on the statistical results of UWB positioning data, identifies the optimal spatial configuration of base stations applicable to formwork scaffold working conditions, verifies the reliability of engineering, compares the differences between PDOP indicators and actual accuracy performance, and conducts theoretical clarification and empirical correction of the limitations of PDOP. Section 5 discusses the practical implications of the research findings in the context of smart construction site safety management and summarizes the core conclusions as well as future research directions.

This study innovates by: (1) conducting a rigorous one-factor controlled experiment to systematically quantify the independent and interactive effects of *H* and *S* on UWB 3D positioning accuracy in simulated formwork scaffolds; (2) quantifying the magnitude of deviation in conventional PDOP assessments under coplanar base station layouts for metal-rich environments; (3) proposing an optimal deployment scheme with RMSE of 0.317 m, which is applicable to practical construction sites.

## 2. Literature Review

### 2.1. Investigation of UWB Base Station Deployment Parameters

Optimizing base station deployment is critical for enhancing positioning accuracy. International studies confirm that the spatial geometry between base stations and mobile terminals plays a crucial role in determining accuracy that can surpass differences between positioning algorithms. Empirical work has demonstrated that deployment parameters like height and spacing directly influence UWB accuracy. Under optimized conditions, such as when H aligns with the target height, positioning error can be reduced by 30% [12]. Furthermore, systematic investigations into base station placement confirm that height, spacing, and overall geometry significantly correlate with ranging errors, and that thoughtful deployment can effectively suppress NLOS errors [13]. In one approach, the spatial configuration was optimized based on geometric volume maximization, verifying that the tetrahedral volume formed by anchors and the tag directly determines the UWB positioning error boundary [14].

The dense metal formwork scaffolds in construction environments disrupt symmetrical base station deployment and induce signal multipath reflection, which distorts parameter effects and necessitates the development of diverse optimization strategies. Among them, one category mainly relies on intelligent optimization algorithms. For instance, one study utilized genetic algorithms to search for the optimal geometric layout of nodes in mining tunnel scenarios [15]. Another category focuses on analyzing independent and interactive effects of key physical parameters through parametric experiments. For example, it was verified through measured data that synergistic optimization of H difference and spacing significantly improves the UWB 3D positioning geometry. Their research indicates that when base station heights are close to target heights and distributed symmetrically, positioning errors in the vertical direction can be significantly reduced [16]. The latter is the route adopted in this study, aiming to provide more direct and interpretable guidance for engineering applications.

However, the systematic application of the aforementioned deployment schemes to the specific context of formwork scaffolds remains limited. Currently, few studies have systematically considered the specific impact of single parameters. Namely, the installation of *H* and *S* base stations in UWB positioning systems, on positioning accuracy within the formwork scaffold structure. In actual formwork scaffolding construction scenarios, different configurations of *H* and spacing alter the coverage range and line-of-sight (LOS) conditions of UWB signals, and this is one of the key factors contributing to fluctuations in positioning accuracy. The formwork scaffold environment is characterized by dense formwork scaffold members, complex spatial structures, and a large dynamic range of personnel, and the occlusion patterns of UWB signals within this environment exhibit both universality and particularity. Many existing studies focus on algorithmic mitigation of NLOS effect, and lack in-depth research on the quantitative relationship between base station deployment parameters and positioning accuracy under the structure of formwork scaffold frames. Therefore, this study aims to address this research gap. By means of rigorous parameter-controlled experiments, it systematically investigates the independent and interactive effects of *H* and *S* on UWB positioning accuracy in simulated formwork scaffolding construction scenarios, and accurately quantifies their optimization effects.

### 2.2. Investigation of UWB Positioning Error Formation Mechanism

To provide a traceable theoretical basis for scenario selection, this study first conducts a systematic analysis of the formation mechanism of UWB positioning errors from both theoretical and literature perspectives. A comparison of WiFi-FTM and UWB measurements at actual construction sites revealed that occlusion and reflection in complex spatial structures cause significant accuracy differences between LOS and NLOS states, indicating that propagation conditions—from unobstructed to severely obstructed—must be fully considered in scenario design [17]. Simulation and empirical studies have shown that the spatial arrangement and height difference in base stations play a decisive role in the GDOP for 3D positioning and vertical errors [6,13]. When base stations and tags are in an approximately coplanar state, the vertical component error is significantly amplified. Further supporting this, a GDOP-based base station optimization method demonstrated that base station spacing and deployment geometry directly influence the mean square error (MSE) and RMSE of positioning, providing theoretical support for constructing different geometric scenarios by adjusting *H* and *S* [18]. Meanwhile, research on UWB positioning in NLOS environments found that multipath reflection and occlusion complexity increase nonlinearly with the spatial variation in construction structures, thereby amplifying positioning errors [19]. Therefore, signal propagation complexity must be a key criterion for classifying working conditions.

**Figure 1:**
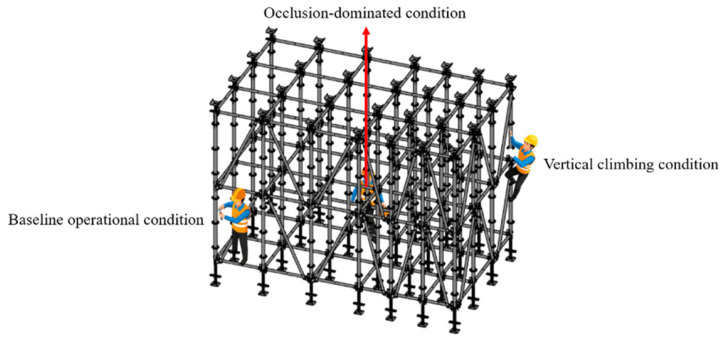
Three operational modes.

Based on the aforementioned research findings, this study correlates typical movement patterns in formwork scaffold operations with channel characteristics. Three representative working conditions, high-altitude fixed-point, vertical penetration, and base-level horizontal, were defined, which provide theoretical support and parameter basis for subsequent experiments, while the three operational modes are shown in Figure 1. High-altitude fixed-point operations reflect working conditions with significant vertical occlusion and *Z*-axis error sensitivity. Vertical penetration operations were characterized by personnel movement between multi-layered formwork scaffolds and time-varying dynamic NLOS states of signals. Base-level horizontal operations correspond to stable scenarios with advantageous LOS conditions, where errors are primarily influenced by horizontal geometric layouts. To systematically reproduce these characteristics in experiments, this study selects *H* of 1.5 m, 2.0 m, and 2.5 m, and *S* of 1.0 m, 1.5 m, and 2.0 m as discrete parameter values within the scope of engineering feasibility, which, respectively, represent height configurations corresponding to low-level occlusion, mid-level balance, and high-level reflection, as well as deployment densities of dense, moderate, and sparse.

### 2.3. Investigation of the Limitations of PDOP

PDOP is a key indicator for evaluating the impact of the spatial geometric configuration of base stations on positioning accuracy in positioning systems, and its value directly reflects the quantitative relationship between spatial geometric distribution and positioning accuracy [20]. The derivation of PDOP starts from the linearized model of GNSS pseudorange observation equations. The covariance matrix of the user state solution is obtained through least squares estimation, and PDOP is ultimately defined as the square root of the trace of the position component of this matrix. Let the matrix $G={\left(H^{T}H\right)}^{-1}$, where Equation (1) is an n×3 dimensional geometric matrix, and each row of (1) consists of the negative value of the unit direction vector from the fixed point to the *i-*th base station. where *r*_i_ denotes the Euclidean distance between the target fixed point and the *i*-th base station

(1) $$H=\left(\begin{array}{ccc} \frac{{x^{\prime }}_{1}-x^{\prime }}{r_{1}} & \frac{{y^{\prime }}_{1}-y^{\prime }}{r_{1}} & \frac{{z^{\prime }}_{1}-z^{\prime }}{r_{1}}\\ \frac{{x^{\prime }}_{2}-x^{\prime }}{r_{2}} & \frac{{y^{\prime }}_{2}-y^{\prime }}{r_{2}} & \frac{{z^{\prime }}_{2}-z^{\prime }}{r_{2}}\\ \vdots & \vdots & \vdots \\ \frac{{x^{\prime }}_{i}-x^{\prime }}{r_{i}} & \frac{{y^{\prime }}_{i}-y^{\prime }}{r_{i}} & \frac{{z^{\prime }}_{i}-z^{\prime }}{r_{i}} \end{array}\right)$$

Among the parameters, (*x_i_*, *y_i_*, *z_i_*) denotes the coordinates of base station *I*. (*x*′, *y*′, *z*′) denotes the coordinates of a fixed point, and *r_i_* denotes the distance from the fixed point to base station *i*. With these parameters, the following calculation can be performed in Equation (2).

(2) $$PDOP=\sqrt{tr(G)}=\sqrt{G_{xx}+G_{yy}+G_{zz}}$$

PDOP can be further refined into horizontal dilution of precision (HDOP) and vertical dilution of precision (VDOP) in Equations (3) and (4).

(3) $$HDOP=\sqrt{G_{xx}+G_{yy}}$$

(4) $$VDOP=\sqrt{G_{zz}}$$

Mathematically, the design matrix *Q* remains nonsingular only when the anchors form a strictly 3D non-coplanar geometry. If all anchors are deployed at the exact same height (coplanar), the *Z*-axis column vectors in *Q* become linearly dependent. This causes severe rank deficiency and near-singularity. Consequently, the diagonal element corresponding to the *Z*-axis in the inverse matrix (*Q^T^Q)*^−1^ approaches infinity. This mathematical singularity directly triggers an extreme spike in VDOP, while HDOP may remain relatively stable if the horizontal XY geometry is sufficient.

As demonstrated in prior research, a coplanar layout of UWB base stations can result in extremely high PDOP values, which are associated with amplified *Z*-axis positioning errors and reduced vertical geometric observability under such setups [13]. To examine this phenomenon, the spatial configuration of base stations in this study adopts a coplanar layout, aiming to analyze the behavior of PDOP and its correspondence with positioning accuracy under coplanar deployment conditions.

Although a non-coplanar anchor layout theoretically reduces VDOP and improves *Z*-axis accuracy, this study deliberately selected a coplanar layout as the experimental baseline to simulate the worst-case engineering scenario prevalent in formwork construction. In actual underground scaffold projects, anchors are often constrained to specific horizontal layers due to layer-wise assembly processes, cabling limitations, and the need to avoid obstructing workflows. These engineering constraints force the system to operate under severely degraded vertical geometric observability. By demonstrating that engineering-grade accuracy is achievable even under such coplanar constraints solely through the optimization of *H* and *S*, this study validates the robustness of the proposed solution for resource-constrained sites, thereby eliminating the reliance on idealized non-coplanar deployments

## 3. Experiment Design

In confined underground space construction scenarios, communication bottlenecks such as NLOS transmission cause personnel safety information to exhibit “blind zone” characteristics. Currently, reliable engineering real-time positioning for construction workers is recognized as a core scientific issue and a key technical prerequisite for breaking through safety perception blind zones and building safe construction sites. In complex construction undertakings within confined underground spaces, traditional GPS and Bluetooth positioning systems suffer from prominent NLOS effects, severe multipath interference, and enhanced electromagnetic noise coupling. These factors result in nonlinear amplification of signal attenuation and spatiotemporal drift, leading to a sharp degradation in positioning accuracy and robustness, which makes the systems unable to meet the demand for reliable engineering real-time positioning. A stepwise one-factor controlled design was adopted to reflect realistic deployment procedures in construction sites.

To capture the authentic working conditions of underground construction, a real-world excavated station project in Chongqing was selected as the test site. Unlike fully operational sites characterized by chaotic machinery and dynamic clutter, this specific test zone was maintained in a semi-controlled state. This isolation was intentional and critical: it allows us to decouple the specific multipath effects caused by the formwork scaffold structure from random environmental noise (e.g., moving vehicles or uneven tunnel walls). Therefore, the resulting error profiles can be specifically attributed to the scaffold’s steel density and the base station deployment geometry. The test focuses on evaluating the static positioning accuracy baseline of the UWB positioning system within formwork scaffolds. Although the ultimate engineering objective is real-time monitoring, this study deliberately adopts a discretized static measurement protocol. This approach is essential to decouple the geometric multipath effects specific to the scaffold structure from motion-induced artifacts (e.g., Doppler shifts, time synchronization latency). Understanding these static error mechanisms is a prerequisite for robust dynamic algorithm design. This design systematically quantifies how anchor height and horizontal spacing influence positioning accuracy. The findings provide a rigorous theoretical and practical foundation for high-precision personnel tracking in complex environments.

The UWB positioning equipment utilized in this experiment is the UWB Mini 5 series module launched by YCHIOT (Yanchuang Internet of Things). The module employs the STM32G070RBT6 microcontroller as its core processing unit, and integrates components such as the DWM1000 communication module, power management module, LED status indication module, and reset circuit in its peripheral circuit. The positioning principle is the Time-of-Arrival (TOA) method. Under ideal working conditions without significant environmental interference, the equipment achieves a typical ranging accuracy of ±10 cm, which meets the requirements of high-precision distance measurement. For general occluded environments, such as non-dense vegetation occlusion and low-rise building occlusion, the equipment optimizes performance through adaptive signal processing algorithms, and the positioning accuracy can still be controlled within the range of ±30 cm, thus exhibiting strong environmental adaptability. The performance parameters of the UWB Mini5 module are shown in Table 1. During the signal measurement process, the movement of personnel or obstacles causes dynamic fluctuations in signal strength, which may lead to ranging errors and three-dimensional spatial positioning deviations.

Mathematically, the measured distance ${\hat{d}}_{i}$ between the mobile tag and the *i*-*th* base station is modeled as:

(5) $${\hat{d}}_{i}=c\cdot \tau_{p,i}+\varepsilon_{i}$$

where c represents the speed of light, and $\tau_{p,i}$ denotes the signal propagation time. The term $\varepsilon_{i}$ ignifies the composite ranging error, which aggregates system noise, clock drift, and—crucially for this study—the positive bias induced by NLOS propagation and multipath effects caused by the scaffold structure. ased on the trilateration principle, the 3D coordinate estimate (*x*, *y*, *z*) of the tag is determined by solving the spherical intersection equations established by N base stations (in this experiment, N = 4):

(6) $${\left(x-x_{i}\right)}^{2}+{\left(y-y_{i}\right)}^{2}+{\left(z-z_{i}\right)}^{2}={\hat{d}}_{i}^{2},\;i=1,2,\ldots ,N$$

where $\left(x\_i,\;y\_i,\;z\_i\right)$ are the known coordinates of the i−th base station. To solve this non-linear system, the equations are typically linearized into the matrix form AX=b and solved via Least Squares Estimation (LSE) to minimize the sum of squared ranging residuals:

(7) $$X={\left(A^{T}A\right)}^{-1}A^{T}b$$

Although LSE is sensitive to NLOS-induced positive bias, optimizing deployment parameters shifts most signal paths from severe NLOS to Quasi-LOS. Here, Quasi-LOS denotes a propagation state where direct signals partially penetrate sparse structural obstacles, causing milder attenuation and bias than complete NLOS blockage. This transformation better aligns the error distribution with the zero-mean Gaussian assumption required by LSE. This modality ensures that the positioning accuracy is primarily sensitive to the geometric quality of the base station deployment (quantified by GDOP) and the magnitude of the ranging error $\varepsilon_{i}$, thus justifying the necessity of optimizing physical deployment parameters (*H* and *S*) to mitigate geometric dilution and signal obstruction. It is important to note the relationship: GDOP^2^ = PDOP^2^ + TDOP^2^, where TDOP represents Time Dilution of Precision. Because this study focuses strictly on optimizing 3D spatial physical deployment geometry, we isolate the spatial component and utilize PDOP as the primary evaluation metric.

**Figure 2:**
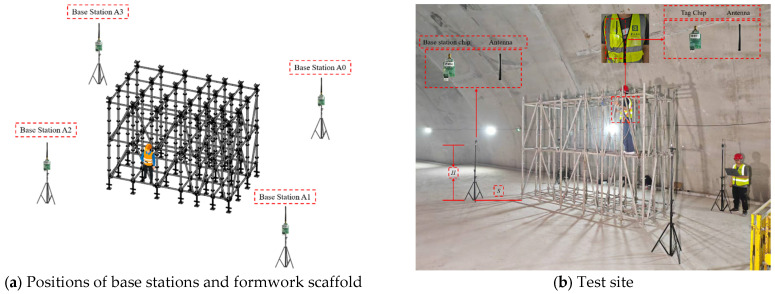
Layout of Test Scenarios.

The formwork scaffold system constructed in this experiment employs a socket-and-disc buckle-type steel pipe formwork scaffold system, featuring a double-layer arrangement with an overall dimension of 4.5 m in length, 1.8 m in width, and 3 m in height. The horizontal members of the formwork scaffold utilize two specifications, with lengths of 600 mm and 900 mm, respectively. The vertical poles follow a unified specification of 1500 mm. As shown in Figure 2, after the completion of formwork scaffold erection, one UWB base station is deployed at each of the four corners of the formwork scaffold, numbered A0–A3, to establish a planar reference coordinate system. Among the parameters, Parameter *H* represents the base station deployment height, defined as the vertical distance from the top of the tripod (i.e., the base station installation plane) to the ground. Parameter *S* represents the horizontal deployment spacing of the base stations, defined as the horizontal straight-line distance from the center of the tripod to the center of the bottom support at the nearest corner of the formwork scaffold. The direction of this line forms a 45° angle with the edge of the adjacent formwork scaffold to ensure consistent spatial geometric relationships among all base stations. Base Station A0 is connected to a host computer via a USB interface, responsible for data transmission and system synchronization.

During the experiment, test subjects wear a UWB tag on the chest and move freely within the space defined by the formwork scaffold. Through the tag signals received by each base station, the coordinate data (*X*′, *Y*′, *Z*′) of the test subjects in three-dimensional space can be calculated and acquired in real time, thus capturing the coordinate data at critical nodes of typical movement trajectories. This static discretization allows for the precise quantification of environmental interference at specific operational points, which is necessary to calibrate future dynamic tracking models.

**Figure 3:**
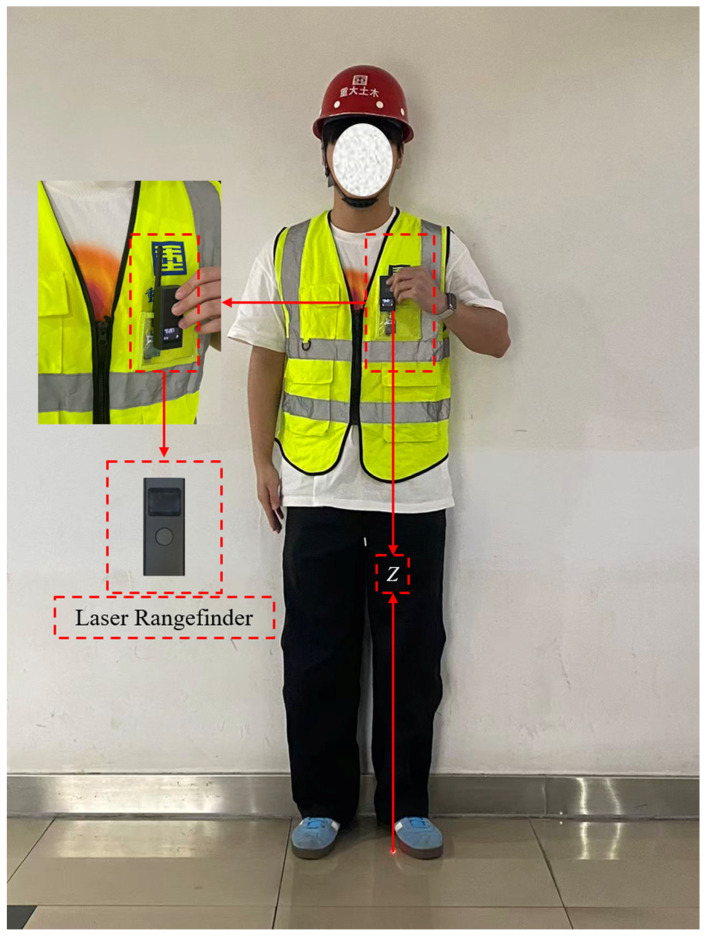
Schematic Diagram of Relative Position Between Laser Rangefinder and Tag Chip.

To ensure the accuracy of the spatial coordinate data of the control group, on-site measurement is performed by the test personnel using a handheld laser rangefinder, as illustrated in Figure 3. The vertical projection (*Z*) was precisely captured via a laser rangefinder. Subsequently, based on the projected spot formed by the laser beam on the ground, the horizontal position of this spot in the preset 2D coordinate system is measured using a calibrated steel tape measure, and its *X*-coordinate and *Y*-coordinate are obtained, respectively. To minimize manual measurement errors, closed-loop geometric verification was performed for all control points prior to UWB data collection. Through this method, the 3D coordinates (*X*, *Y*, *Z*) of the measurement point for the control group are finally determined, which serve as high-precision reference true values for subsequent evaluation of the accuracy and reliability of the UWB positioning system. To quantify data errors, RMSE is adopted as the primary evaluation indicator. Additionally, comprehensive statistical parameters—including Mean, Median, Standard Deviation (SD), and 95% Confidence Intervals (CI)—alongside paired *t*-tests, are utilized to rigorously evaluate the statistical significance and spatial consistency of the positioning deviations. All test subjects provided informed consent prior to the experiment, and the study was conducted in compliance with the ethical guidelines of Chongqing University.

(8) $$RMSE=\sqrt{\frac{1}{N}\sum_{i=1}^{N}\left[{\left(x_{i}-x_{true}\right)}^{2}+{\left(y_{i}-y_{true}\right)}^{2}+{\left(z_{i}-z_{true}\right)}^{2}\right]}$$

## 4. Data and Error Analysis

### 4.1. Parametric Control Testing

To quantify positioning errors induced strictly by spatial geometry and environmental multipath—rather than by time-synchronization latency or Doppler shifts inherent in continuous movement—this study adopted a discretized static measurement protocol. We mapped the three dynamic operational modes (High-altitude, Vertical penetration, Base-level horizontal) defined in Section 2.2 into 10 representative static checkpoints. These points were spatially selected to capture the ‘worst-case’ and ‘typical’ geometric relations occurring during actual worker movements. For instance, checkpoints for the ‘Vertical penetration’ mode were set at varying heights within the dense scaffold matrix to simulate the critical signal occlusion experienced during climbing. At each checkpoint, a multi-epoch static observation protocol (5 sessions per point) was executed to filter random timing noise and obtain convergent steady-state estimates. Five independent observation sessions were recorded for each checkpoint, with outliers filtered to obtain a convergent steady-state position estimate. With a step size of 0.5 m, a parametric control approach is employed: the *H* is fixed while the *S* is varied, and conversely, *S* is fixed while *H* is adjusted. This design aims to systematically investigate the effects of *H* and *S* on UWB positioning accuracy.

**Figure 4:**
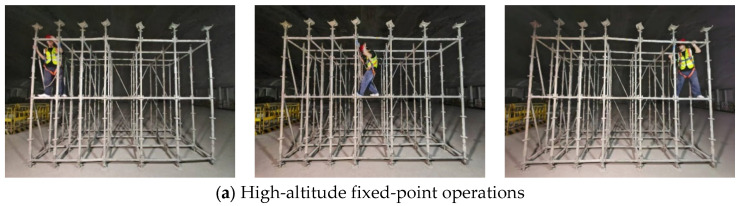

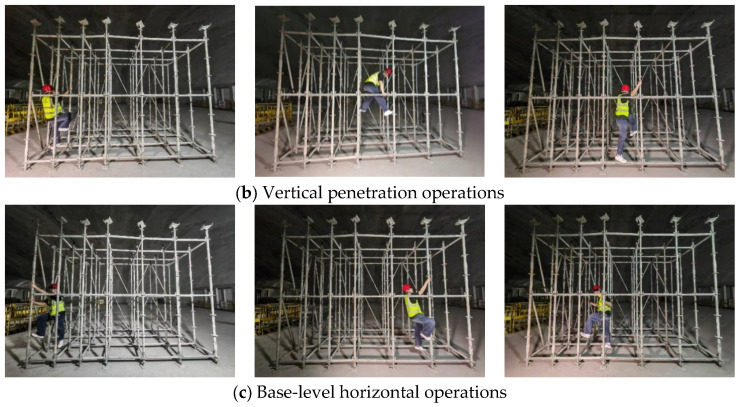
Schematic of Worker Activity Modes on the Formwork Scaffold.

Figure 4 systematically illustrates personnel activities under three core operation modes and emulates typical high-frequency dynamic behaviors of construction workers in complex formwork scaffold structures, including static construction operations, vertical climbing states, and horizontal movement states. This experimental design is intended to accurately quantify the influence mechanisms of key deployment parameters on personnel spatial positioning accuracy by simulating real construction environments. Research findings will provide critical data support and theoretical basis for optimizing positioning systems in complex construction environments, such as formwork scaffolds characterized by NLOS and strong occlusion.

### 4.2. Error Source Discussion

To ensure the reliability of the experimental data collected in Section 4.1, this study systematically analyzed the primary sources of error affecting the positioning system. These errors are categorized into systematic errors, environmental errors, and measurement errors. Table 2 summarizes the primary sources and quantitative impacts of these errors.

To mitigate the aforementioned errors and ensure experimental rigor, this study implemented the following specific strategies:

Systematic Error Mitigation: A time-domain statistical filtering approach was adopted. For each coordinate measurement point, a multi-epoch static observation protocol (5 sessions per point) was executed to filter random timing noise and obtain convergent steady-state estimates.

Environmental Error Mitigation: A worst-case baseline strategy was established to quantify steel member interference. The experiment explicitly selected *H* = 1.5 m and *S* = 1.0 m as the initial parameter set to establish a validity boundary:

*H* = 1.5 m was selected to align with the typical chest height where workers wear tags. Crucially, this height creates a grazing incidence path geometrically coplanar with the dense middle-layer crossbars, thereby exposing the maximum potential environmental occlusion.

*S* = 1.0 m was determined as the operational limit for high-density deployment, as preliminary field tests indicated that spacing configurations below 0.5 m resulted in signal collision and synchronization failures. By anchoring the experiment in these extreme conditions, the study establishes a solid reference baseline for evaluating the error suppression effects of subsequent parameter variations.

It is noteworthy that the determined optimal spacing of *S* = 1.5 m exhibits a strong correlation with the structural dimensions of the formwork scaffold used in this experiment (standard bay width of 1.8 m). This suggests that the optimal deployment spacing may be ratio-dependent on the scaffold’s structural grid, warranting further verification with varying bay widths. Setting *S* slightly narrower than the scaffold’s bay dimension allows the base stations to maintain LOS corridors through the gaps in the steel matrix. Conversely, an arbitrary increase in spacing (e.g., to 2.0 m) might align the signal path directly with oblique steel members, exacerbating occlusion. Therefore, for varying scaffold specifications in engineering practice, we recommend establishing *S* based on the scaffold’s specific grid unit (approximately 0.8–1.0 times the bay width) rather than applying a fixed metric.

Measurement Error Mitigation: Strict geometric constraint verification was performed. Before data collection, closed-loop geometric verification was conducted for all control points, and coordinates were cross-validated using calibrated steel tape measures to minimize manual operation deviations.

### 4.3. Analysis of Horizontal Spacing on Positioning Accuracy

To investigate how variations in the *S* influence UWB positioning errors under a fixed *H* = 2.0 m, this section systematically analyzes the characteristics of positioning errors under different *S* values (1.0 m, 1.5 m, 2.0 m). The objective is to determine the effect of these deployment parameters on the spatial geometry of the signal propagation.

**Figure 5:**
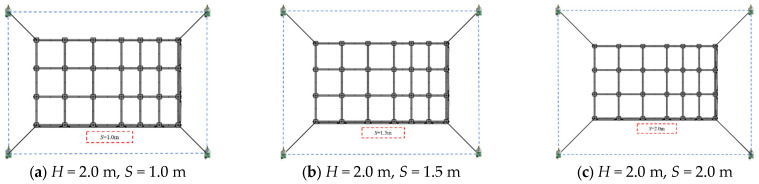
Spatial configurations for different *S* under fixed base station heights (*H*).

Figure 5 illustrates the spatial configurations of the UWB positioning system and the support frame corresponding to different *S* when *H* is fixed.

**Figure 6:**
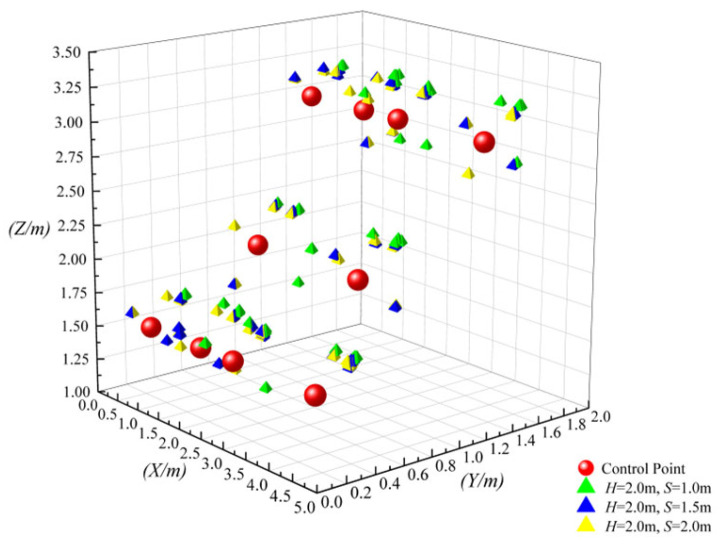
Scatter plot of 3D coordinates with variations in *S* under fixed *H*.

Figure 6 illustrates the scatter distributions of UWB positioning 3D coordinates corresponding to different *S* when *H* is fixed.

As observed in Figure 6, when *S* = 1.0 m, scatters exhibit significant spatial dispersion in the *X*, *Y*, and *Z* dimensions, with a large number of scatters deviating from the core region of the measured reference true values. This dispersion stems from the suboptimal geometry under narrow spacing. The dense scaffold members severely occlude UWB signals, inducing prominent NLOS interference that degrades positioning consistency. When *S* increases to 1.5 m, a stepwise narrowing of the scatter dispersion range occurs, with most scatters rapidly converging around the reference true values and a substantial improvement in spatial matching degree. This indicates that increasing the spacing significantly reduces direct signal occlusion by formwork scaffold structures, effectively suppressing NLOS interference. When *S* further increases to 2.0 m, only a marginal improvement in scatter aggregation is observed. Compared with *S* = 1.5 m, the tendency of scatters to move toward the core region becomes less pronounced, with only minor convergence of a few edge scatters. The visual difference between the two is far smaller than that between *S* = 1.0 m and *S* = 1.5 m, indicating that the contribution of further increasing spacing to spatial configuration optimization has entered a saturation phase.

**Figure 7:**
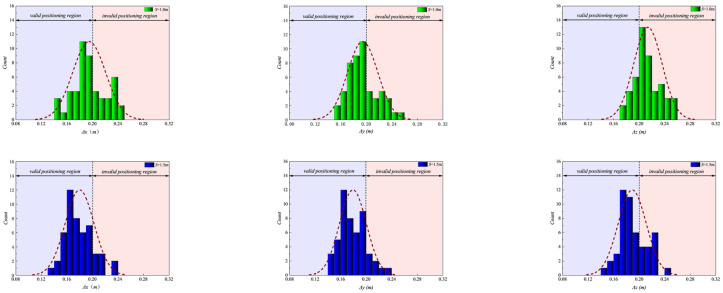

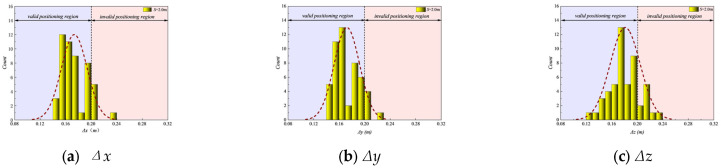
The influence of *S* on UWB positioning errors under fixed *H*.

Figure 7 shows the distribution of UWB positioning errors for different *S*, with *H* fixed. Referring to the technical code for safety of working at height of building construction [21], the single-axis positioning error threshold was established at 0.2 m, striking a balance between hardware limitations and engineering safety requirements. Although the nominal accuracy of the deployed UWB Mini5 module is ±0.1 m, signal degradation due to multipath effects in complex high-formwork environments necessitates a realistic tolerance margin. Consequently, a conservative threshold of 0.2 m was adopted. From a safety perspective, this resolution, approximately one-third of an average human stride, provides sufficient granularity for the deployment of virtual geofences. It ensures an adequate safety buffer for edge protection while mitigating warning failures caused by positioning drift. We acknowledge that a 0.2 m single-axis limit can yield a maximum 3D localization error of roughly 0.35 m. This remains strictly within the 0.5 m safe boundary. A larger single-axis threshold, such as 0.3 m, would produce a maximum 3D error of 0.52 m. This exceeds the reliable geofencing limit. Therefore, 0.2 m provides the optimal safety margin without demanding impossible hardware precision.

Figure 7 illustrates the dynamic evolution of the error probability density function for single-axis positioning (Δ*x*, Δ*y*, Δ*z*) under fixed height conditions (*H* = 2.0 m). The distribution morphology undergoes a distinct transition: at the narrow spacing of *S* = 1.0 m, the histogram exhibits a dispersed, heavy-tailed profile with a significant portion extending into the invalid positioning region (>0.2 m), indicating a high frequency of large deviations. As the spacing expands to *S* = 1.5 m, the distribution undergoes a sharp leftward shift and narrowing process, where the scattered tail is effectively truncated and the peak concentration aligns strictly within the valid positioning region (<0.2 m), reflecting a stabilization of signal quality. Finally, the distribution at *S* = 2.0 m largely overlaps with the 1.5 m profile, visually confirming that the error suppression capability has reached a saturation plateau, with no further significant contraction in the error spread.

Table 3 presents the point-by-point raw data. This illustrates the spatial variance across the scaffold. Table 4 summarizes the comprehensive statistical parameters. These parameters include the Mean, Median, SD, and 95% CI. Increasing the *S* from 1.0 m to 1.5 m significantly improves accuracy. The mean RMSE_3D_ decreases from 0.3473 m to 0.3172 m. The SD remains remarkably narrow at roughly 0.033 m. This indicates high system stability across all test points. The 95% CI for *S* = 1.5 m is [0.2937, 0.3407] m. This upper bound sits strictly below the 0.35 m 3D safety threshold. To verify these improvements, we performed a paired *t*-test. The accuracy gain from *S* = 1.0 m to *S* = 1.5 m is statistically significant (*p* < 0.001). Increasing *S* further from 1.5 m to 2.0 m yields a mean RMSE_3D_ of 0.3059 m. This difference is also statistically significant (*p* < 0.001) due to highly consistent measurements. However, the absolute mean improvement is only 0.011 m. This marginal physical gain confirms a phase of diminishing returns. Therefore, *S* = 1.5 m offers the optimal balance between positioning accuracy and deployment footprint.

### 4.4. Analysis of Base Station Height on Positioning Accuracy

By increasing the height *H*, this study aims to quantify the mechanism by which vertical spatial diversity mitigates these coplanar occlusion effects and optimizes the single-axis error distribution.

**Figure 8:**
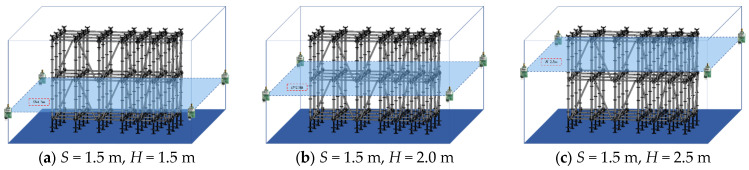
Spatial configurations for different *H* under fixed *S*.

Figure 8 illustrates the spatial configurations of the UWB positioning system and the formwork scaffold corresponding to different base station heights *H* when the *S* is fixed.

**Figure 9:**
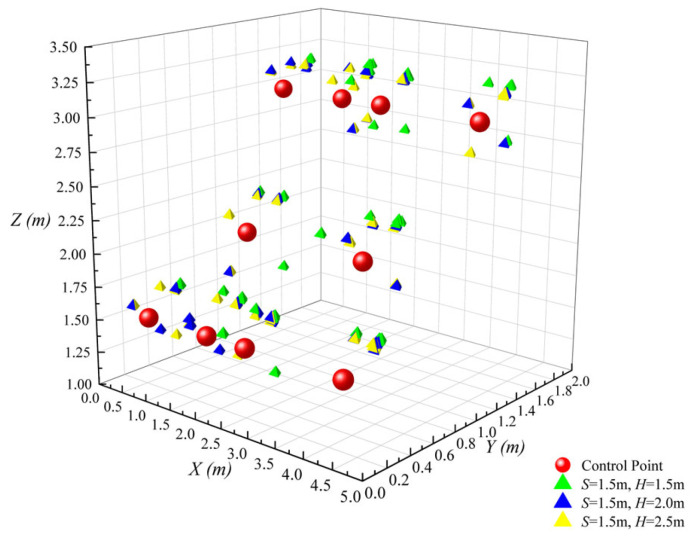
Scatter plot of 3D coordinates with variations in *H* under fixed *S*.

Figure 9 illustrates the scatter distributions of UWB positioning 3D coordinates corresponding to different base station heights *H* when the *S* is fixed.

As indicated in Figure 9, when *H* = 1.5 m, the spatial dispersion of scatters in the vertical direction (*Z*-axis) is significantly stronger than that in the horizontal directions (*X* and *Y* axes): scatters in the *Z*-axis generally deviate from the core region of true values by more than 0.2 m, while scatters in the *X* and *Y* axes exhibit relatively smaller deviation magnitudes. This relates directly to the middle-layer horizontal scaffold members. Signals from low-height base stations suffer severe vertical occlusion. Unlike horizontal paths, vertical paths lack sufficient space for signal diffraction, drastically amplifying *Z*-axis NLOS effects. When *H* increases to 2.0 m, scatters show three-dimensional coordinated convergence: while the dispersion range in the *X* and *Y* axes narrows, scatters in the *Z*-axis almost completely align with the true value region. This indicates that this height precisely avoids the occlusion threshold of the main horizontal members of the formwork scaffold, and the vertical signal propagation path is fully optimized. When *H* further increases to 2.5 m, scatters exhibit *Z*-axis-specific dispersion: the *X* and *Y* axes still maintain high aggregation, but some scatters in the *Z*-axis deviate from the true values. This is presumably due to extended signal propagation paths caused by excessively high base stations, where reflections from top members of the formwork scaffold and environmental noise introduce additional multipath interference, offsetting the mitigating effect of increased height on occlusion.

**Figure 10:**
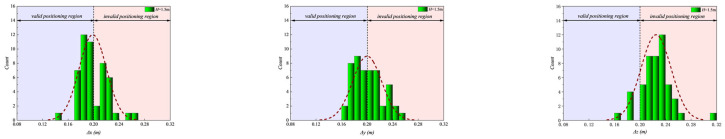

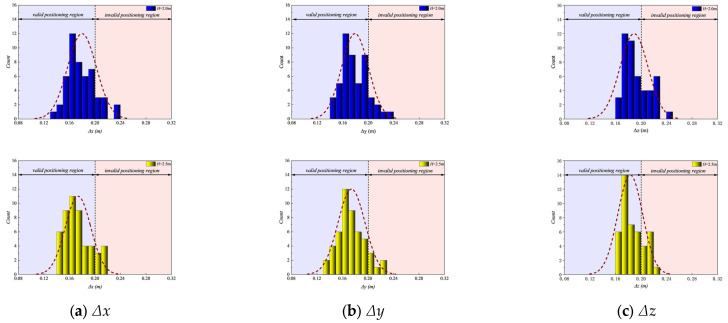
The Influence of *H* on UWB Positioning Errors Under Fixed *S*.

Following the establishment of the 0.2 m safety threshold based on the horizontal spacing analysis (Figure 7), Figure 10 extends this rigorous evaluation to the *H*. Given that the scaffold’s horizontal members create severe coplanar occlusion, the *Z*-axis is theoretically the most vulnerable dimension. Therefore, applying the identical 0.2 m criterion serves as a critical stress test for vertical geometric stability. The objective is to determine whether optimizing H can effectively compress the historically higher vertical errors into the valid engineering domain defined in the previous section, ensuring isotropic safety compliance without relaxing tolerance standards.

Figure 10 depicts the probability density evolution of positioning errors under fixed spacing (*S* = 1.5 m), examining adherence to the established safety limit. A distinct compliance failure is observed in the Δ*z* distribution at *H* = 1.5 m, where the histogram exhibits a flattened, dispersive profile with a substantial probability mass breaching the invalid positioning region boundary, visually quantifying the severity of the coplanar ranging defect. However, elevating to *H* = 2.0 m induces a dramatic spectral retraction, where the error distribution—particularly along the critical Z-axis—reconfigures into a compact, Gaussian-like peak strictly confined within the valid positioning region. This sharp morphological transition visually confirms that the optimized height successfully eliminates the vertical accuracy bottleneck, aligning the system’s vertical performance with the safety benchmarks previously achieved in the horizontal dimensions.

Table 5 lists the point-by-point positioning errors for height variations. Table 6 provides the corresponding statistical summary. Elevating the anchors *H* from 1.5 m to 2.0 m dramatically optimizes vertical performance. The mean RMSE_3D_ decreases from 0.3615 m to 0.3171 m. The *Z*-axis error reduction primarily drives this improvement. The SD stays extremely low at 0.0328 m. The 95% CI for *H* = 2.0 m is [0.2936, 0.3406] m. This guarantees stable, sub-0.35 m accuracy across the entire workspace. A paired *t*-test confirms that this error reduction is highly significant (*p* < 0.001). This optimal height successfully mitigates the coplanar occlusion caused by middle-layer scaffold members. Further elevating the anchors to *H* = 2.5 m reduces the mean RMSE_3D_ to 0.3070 m. Although this reduction is statistically significant (*p* < 0.001), the absolute mean improvement is merely 0.010 m. This mirrors the saturation trend observed in the horizontal spacing. It proves that excessive deployment height is physically unnecessary. Thus, *H* = 2.0 m effectively resolves vertical occlusion with optimal resource efficiency.

With a mean RMSE_3D_ of 0.317 m, a remarkably low SD of 0.033 m, and a 95% CI strictly bounded below 0.35 m, the system exhibits optimal positioning consistency and stability. This statistically validated baseline provides a rigorous quantitative foundation for future dynamic tracking research in complex construction environments

### 4.5. Verification of PDOP Limitations

This study selected two fixed points, P1 (0.80, 1.30, 1.75) and P2 (3.50, 1.50, 2.95), and calculated HDOP, VDOP, and PDOP under different deployments in study.

Through the analysis of Table 7 and Table 8, the following conclusions are drawn. First, in all testing scenarios of both tables, values of HDOP remain stable within the range of 5 to 7. These relatively small and stable HDOP values indicate that, under different deployment parameters, the horizontal positioning accuracy of the system is reliable and acceptable. Second, in sharp contrast to the stable HDOP, values of VDOP exhibit significant fluctuations on an order of magnitude and abnormally high values. These abnormally high VDOP values indicate that vertical geometric observability constitutes the primary sensitivity direction of the positioning system, and that *Z*-axis accuracy is more susceptible to geometric degradation than horizontal components.

The fundamental cause of this phenomenon lies in the coplanar layout of base stations. When heights of multiple base stations are very close, their geometric distribution intensity in the zenith direction weakens. From the receiver’s perspective, base stations are concentrated within a small elevation angle range overhead, rendering the system unable to precisely distinguish small differences in height from which signals originate. This geometric structural weakness manifests as a sharp increase in VDOP values, which elevates the overall PDOP. The relationship between PDOP and actual 3D RMSE is mathematically non-linear. For example, when anchor height decreases from 2.0 m to 1.5 m (coplanar), the calculated PDOP spikes exponentially from 41.5 to 114.0 (a 174% increase). If the correlation were linear, the RMSE should similarly nearly triple. However, the actual RMSE only increases from 0.317 m to 0.361 m (a mere 14% increase). This extreme quantitative divergence proves that PDOP heavily overestimates vertical geometric degradation while failing to capture actual physical ranging stability.

## 5. Conclusions

This study experimentally validated a practical optimization strategy for UWB base station deployment to improve positioning accuracy in formwork scaffold environments subject to severe NLOS interference. A one-factor controlled experimental design was employed to quantify the independent effects of *H* and *S*, and the influence of deployment geometry was interpreted through integrated error evaluation, spatial geometry modeling, and DOP analysis.

The results confirm that deployment geometry plays a decisive role in UWB positioning performance. For the specific 1.8 m bay width tested, the optimal spacing was 1.5 m (a ratio of 0.8). However, this remains a site-specific hypothesis. A key limitation of this study is the reliance on a single structural geometry. Due to logistical and safety constraints in active underground sites, testing diverse geometries was unfeasible. Future research must validate this proposed ratio dependency across varying scaffold configurations. Specifically, the optimal spacing is approximately 0.8 times the bay width (e.g., *S* = 1.5 m for a 1.8 m grid), combined with an anchor height of *H* = 2.0 m. Under this configuration, the system achieved the best overall performance, with a minimum 3D RMSE of 0.317 m and more than 80% of single-axis errors falling within the engineering-valid region. Increasing *H* enhances vertical signal visibility and LOS propagation, while an appropriate *S* suppresses inter-anchor interference and mitigates multipath distortion. Excessive spacing, however, weakens geometric constraints and degrades lateration accuracy, indicating that vertical and horizontal parameters must be jointly optimized.

The study further shows that the conventional PDOP metric does not linearly reflect actual positioning accuracy under coplanar or near-coplanar deployments in metal-rich environments. A deployment evaluation approach that jointly considers geometric configuration and environmental interference is therefore more appropriate for practical engineering applications. Theoretically, deploying eight nodes at varied heights (e.g., 2.0 m and 0.5 m) would resolve vertical rank deficiency and mitigate VDOP spikes. Practically, layer-wise assembly and cabling constraints in active underground scaffolds restrict anchors to a single horizontal plane. Our minimal 4-node coplanar setup intentionally simulates this worst-case engineering reality. The proposed low-redundancy deployment scheme provides a balanced solution between positioning accuracy and installation cost, demonstrating feasibility for real-world construction safety monitoring. Future work will extend the static baseline established in this study toward dynamic tracking and sensor fusion to enhance robustness under time-varying NLOS conditions.

## Figures and Tables

**Table 1:** Performance specifications of UWB Mini5 module.

Parameter	Specification
Power Interface	Micro-USB (5.0 V)
Communication Interface	Micro-USB (5.0 V)/Serial Port (3.3 V TTL)
Data Rate	110.0 Kb/s~6.8 Mb/s
Operating Frequency	3.5 GHz~6.5 GHz
Transmission Power Density	−35.0 dbm/MHz~−62.0 dbm/MHz
Positioning Accuracy	±10.0 cm
Communication Range	80.0 m

**Table 2:** Analysis of error sources.

Error Category	Primary Source	Quantitative Impact
Systematic Error	Internal clock drift and synchronization jitter of UWB modules.	<±5 ns
Environmental Error	Multipath delay and NLOS propagation induced by signal reflection from dense steel members.	<0.1 m
Measurement Error	Manual operation deviation of the laser rangefinder during control point data acquisition.	<±2 mm

**Table 3:** Single-axis and 3D Error Statistics for Different *S* (*H* = 2.0 m).

Deployment	RMSE*_x_* (m)	RMSE*_y_* (m)	RMSE*_z_* (m)
*H* = 2.0 m, *S* = 1.0 m	0.1868	0.1855	0.2126
	0.2067	0.2096	0.2222
	0.2304	0.2314	0.2492
	0.1798	0.1740	0.2020
	0.1896	0.1912	0.2015
	0.1827	0.1805	0.1947
	0.2015	0.2001	0.2164
	0.2215	0.2225	0.2336
	0.1729	0.1716	0.1843
	0.1826	0.1826	0.1904
*H* = 2.0 m, *S* = 1.5 m	0.1691	0.1691	0.1820
	0.1892	0.1909	0.1998
	0.2112	0.2090	0.2211
	0.1611	0.1593	0.1686
	0.1717	0.1704	0.1807
	0.1689	0.1697	0.1778
	0.1909	0.1911	0.1967
	0.2092	0.2086	0.2198
	0.1594	0.1582	0.1689
	0.1708	0.1690	0.1794
*H* = 2.0 m, *S* = 2.0 m	0.1636	0.1632	0.1756
	0.1823	0.1838	0.1923
	0.2050	0.2017	0.2159
	0.1550	0.1516	0.1633
	0.1654	0.1622	0.1758
	0.1623	0.1628	0.1733
	0.1826	0.1830	0.1910
	0.2018	0.2027	0.2138
	0.1533	0.1507	0.1621
	0.1640	0.1611	0.1749

**Table 4:** Statistical parameters of 3D positioning errors for different S (H = 2.0 m).

*S* (m)	Mean RMSE*_x_* (m)	Mean RMSE*_y_* (m)	Mean RMSE*_z_* (m)	Mean RMSE_3D_ (m)	Media (m)	SD (m)	95% CI (m)
1.0	0.1955	0.1949	0.2107	0.3473	0.3374	0.0341	[0.3229, 0.3717]
1.5	0.1801	0.1795	0.1895	0.3171	0.3012	0.0328	[0.2937, 0.3407]
2.0	0.1735	0.1723	0.1838	0.3059	0.2906	0.0327	[0.2825, 0.3293]

**Table 5:** Single-axis and 3D error statistics for different *H* (*S* = 1.5 m).

Deployment	RMSE*_x_* (m)	RMSE*_y_* (m)	RMSE*_z_* (m)
*S* = 1.5 m, *H* = 1.5 m	0.1922	0.1920	0.2192
	0.2054	0.2123	0.2371
	0.2318	0.2338	0.2536
	0.1829	0.1798	0.2104
	0.1889	0.1939	0.2283
	0.1899	0.1886	0.2057
	0.2053	0.2102	0.2282
	0.2282	0.2257	0.2501
	0.1766	0.1762	0.2027
	0.1889	0.1917	0.2206
*S* = 1.5 m, *H* = 2.0 m	0.1691	0.1691	0.1820
	0.1892	0.1909	0.1998
	0.2112	0.2090	0.2211
	0.1611	0.1593	0.1686
	0.1717	0.1704	0.1807
	0.1689	0.1697	0.1778
	0.1909	0.1908	0.1967
	0.2092	0.2086	0.2198
	0.1594	0.1582	0.1689
	0.1708	0.1690	0.1794
*S* = 1.5 m, *H* = 2.5 m	0.1665	0.1674	0.1733
	0.1839	0.1847	0.1924
	0.2049	0.2056	0.2139
	0.1533	0.1544	0.1649
	0.1635	0.1649	0.1745
	0.1623	0.1628	0.1733
	0.1831	0.1834	0.1911
	0.2036	0.2049	0.2127
	0.1530	0.1535	0.1638
	0.1626	0.1638	0.1737

**Table 6:** Statistical parameters of 3D positioning errors for different H (S = 1.5 m).

*H* (m)	Mean RMSE*_x_* (m)	Mean RMSE*_y_* (m)	Mean RMSE*_z_* (m)	Mean RMSE_3D_ (m)	Media (m)	SD (m)	95% CI (m)
1.5	0.1990	0.2004	0.2256	0.3615	0.3516	0.0314	[0.3391, 0.3840]
2.0	0.1801	0.1795	0.1895	0.3171	0.3012	0.0328	[0.2937, 0.3407]
2.5	0.1737	0.1746	0.1834	0.3070	0.2917	0.0327	[0.2836, 0.3304]

**Table 7:** Impact of *S* on HDOP, VDOP, PDOP (*H* = 2.0 m).

Confirmation Point	Deployment	HDOP	VDOP	PDOP
P1	*S* = 1.0 m, *H* = 2.0 m	5.9867	58.6702	58.9749
	*S* = 1.5 m, *H* = 2.0 m	5.6113	52.7302	53.0279
	*S* = 2.0 m, *H* = 2.0 m	5.3914	49.2129	49.5074
P2	*S* = 1.0 m, *H* = 2.0 m	5.9609	24.4915	25.2065
	*S* = 1.5 m, *H* = 2.0 m	5.9961	29.4304	30.0350
	*S* = 2.0 m, *H* = 2.0 m	6.3572	36.1252	36.6803

**Table 8:** Impact of *H* on HDOP, VDOP, PDOP (*S* = 1.5 m).

Confirmation Point	Deployment	HDOP	VDOP	PDOP
P1	*S* = 1.5 m, *H* = 1.5 m	5.5312	202.7752	202.8506
	*S* = 1.5 m, *H* = 2.0 m	5.5593	82.2484	82.4361
	*S* = 1.5 m, *H* = 2.5 m	5.9526	27.4252	28.0638
P2	*S* = 1.5 m, *H* = 1.5 m	6.5862	24.2710	25.1488
	*S* = 1.5 m, *H* = 2.0 m	5.9961	29.4304	30.0350
	*S* = 1.5 m, *H* = 2.5 m	5.6477	53.2744	53.5730

## Data Availability

Data available on request from the authors.
